# Incremental value of biomarker combinations to predict progression of mild cognitive impairment to Alzheimer’s dementia

**DOI:** 10.1186/s13195-017-0301-7

**Published:** 2017-10-10

**Authors:** Lutz Frölich, Oliver Peters, Piotr Lewczuk, Oliver Gruber, Stefan J. Teipel, Hermann J. Gertz, Holger Jahn, Frank Jessen, Alexander Kurz, Christian Luckhaus, Michael Hüll, Johannes Pantel, Friedel M. Reischies, Johannes Schröder, Michael Wagner, Otto Rienhoff, Stefanie Wolf, Chris Bauer, Johannes Schuchhardt, Isabella Heuser, Eckart Rüther, Fritz Henn, Wolfgang Maier, Jens Wiltfang, Johannes Kornhuber

**Affiliations:** 1 Department of Geriatric Psychiatry, Central Institute of Mental Health, Medical Faculty Mannheim, University of Heidelberg, Zentralinstitut für Seelische Gesundheit, Quadrat J5, D-68159 Mannheim, Germany; 2 Department of Psychiatry and Psychotherapy, Campus Benjamin Franklin, Charité, Berlin, Germany; 3 Department of Psychiatry and Psychotherapy, Friedrich-Alexander-University of Erlangen-Nuremberg, Nuremberg, Germany; 4 Department of Neurodegeneration Diagnostics, Medical University of Bialystok, Bialystok, Poland; 5 Department of Psychiatry and Psychotherapy, University Medical Center Göttingen, and German Center for Neurodegenerative Diseases (DZNE), Research Site Göttingen, Göttingen, Germany; 6 German Center for Neurodegenerative Diseases (DZNE), Rostock, Germany; 7 Department of Psychosomatic Medicine, University Medicine Rostock, Rostock, Germany; 8 Department of Psychiatry and Psychotherapy, Ludwig-Maximilian-University of Munich, Munich, Germany; 9 Department of Psychiatry, University of Leipzig, Leipzig, Germany; 10 Department of Psychiatry and Psychotherapy, University Medical Center Hamburg, Hamburg, Germany; 11 Department of Psychiatry and Psychotherapy, University of Bonn, Bonn, Germany; 12 German Center for Neurodegenerative Diseases (DZNE), Cologne/Bonn, Germany; 13 Department of Psychiatry and Psychotherapy, Medical Faculty University of Cologne, Cologne, Germany; 14 Department of Psychiatry and Psychotherapy, Technical University of Munich, Munich, Germany; 15 Department of Psychiatry and Psychotherapy, University of Düsseldorf, Düsseldorf, Germany; 16 Center for Psychiatry, Clinic for Geriatric Psychiatry and Psychotherapy Emmendingen and Department of Psychiatry and Psychotherapy, University of Freiburg, Freiburg, Germany; 17 Institute of General Medicine University of Frankfurt, Frankfurt am Main, Germany; 18 Section for Geriatric Psychiatry Research, Department for Psychiatry, University of Heidelberg, Heidelberg, Germany; 19 Department of Medical Informatics, University of Göttingen, Göttingen, Germany; 20 MicroDiscovery GmbH, Berlin, Germany

**Keywords:** Mild cognitive impairment, Biomarkers, Tau, Phospho-tau, Amyloid-beta 42, Hippocampal volume, Prediction, Alzheimer’s dementia

## Abstract

**Background:**

The progression of mild cognitive impairment (MCI) to Alzheimer’s disease (AD) dementia can be predicted by cognitive, neuroimaging, and cerebrospinal fluid (CSF) markers. Since most biomarkers reveal complementary information, a combination of biomarkers may increase the predictive power. We investigated which combination of the Mini-Mental State Examination (MMSE), Clinical Dementia Rating (CDR)-sum-of-boxes, the word list delayed free recall from the Consortium to Establish a Registry of Dementia (CERAD) test battery, hippocampal volume (HCV), amyloid-beta_1–42_ (Aβ42), amyloid-beta_1–40_ (Aβ40) levels, the ratio of Aβ42/Aβ40, phosphorylated tau, and total tau (t-Tau) levels in the CSF best predicted a short-term conversion from MCI to AD dementia.

**Methods:**

We used 115 complete datasets from MCI patients of the “Dementia Competence Network”, a German multicenter cohort study with annual follow-up up to 3 years. MCI was broadly defined to include amnestic and nonamnestic syndromes. Variables known to predict progression in MCI patients were selected a priori. Nine individual predictors were compared by receiver operating characteristic (ROC) curve analysis. ROC curves of the five best two-, three-, and four-parameter combinations were analyzed for significant superiority by a bootstrapping wrapper around a support vector machine with linear kernel. The incremental value of combinations was tested for statistical significance by comparing the specificities of the different classifiers at a given sensitivity of 85%.

**Results:**

Out of 115 subjects, 28 (24.3%) with MCI progressed to AD dementia within a mean follow-up period of 25.5 months. At baseline, MCI-AD patients were no different from stable MCI in age and gender distribution, but had lower educational attainment. All single biomarkers were significantly different between the two groups at baseline. ROC curves of the individual predictors gave areas under the curve (AUC) between 0.66 and 0.77, and all single predictors were statistically superior to Aβ40. The AUC of the two-parameter combinations ranged from 0.77 to 0.81. The three-parameter combinations ranged from AUC 0.80–0.83, and the four-parameter combination from AUC 0.81–0.82. None of the predictor combinations was significantly superior to the two best single predictors (HCV and t-Tau). When maximizing the AUC differences by fixing sensitivity at 85%, the two- to four-parameter combinations were superior to HCV alone.

**Conclusion:**

A combination of two biomarkers of neurodegeneration (e.g., HCV and t-Tau) is not superior over the single parameters in identifying patients with MCI who are most likely to progress to AD dementia, although there is a gradual increase in the statistical measures across increasing biomarker combinations. This may have implications for clinical diagnosis and for selecting subjects for participation in clinical trials.

## Background

Slowly progressive mild cognitive impairment (MCI) with insidious onset often results in neurodegenerative dementia, e.g., dementia due to Alzheimer’s disease (AD). A current plausible model for the development of AD suggests a temporal order of pathological brain changes; amyloid deposition occurs early in the disease, but may not directly cause clinical symptoms and is believed to trigger neuronal injury and loss [1, 2]. Neuronal and synaptic losses are key determinants of cognitive impairment, which are accompanied by brain atrophy on magnetic resonance imaging (MRI) [3, 4]. Thus, the pathological cascade in AD is regarded as a two-stage, slowly progressive process in which amyloidosis and neuronal injury (tauopathy and neurodegeneration) are largely sequential rather than simultaneous processes [1, 2].

The predementia phase of AD is characterized clinically by MCI [5], and this is accompanied by biochemical changes in the brain reflected in the cerebrospinal fluid (CSF) [6, 7] as well as in brain morphology on MRI [8, 9]. Specifically, impairments in delayed free-recall measures from episodic memory tasks [10–12], reduced hippocampal volumes [12–15], decreased CSF levels of amyloid-beta_1–42_ (Aβ42; a marker of amyloid mismetabolism), and elevations in tau and phosphorylated tau (p-Tau) protein (markers of axonal damage and neurofibrillary tangles) [6, 14, 16–19] are the best established predictive biomarkers of AD dementia in patients with MCI [20].

When analyzing the relation between these biomarkers and progression to AD dementia or cognitive decline in MCI patients, most previous studies have either determined the strength of association between biomarkers at baseline and cognitive/functional decline at follow-up (continuous variables), or how well biomarker levels at baseline were able to predict a diagnosis of AD dementia at follow-up (dichotomous variable). The differential predictive power of different biomarkers or combinations of biomarkers was mainly evaluated descriptively, not by statistical testing.

A wealth of data exists to show that the brain changes on MRI are related to CSF biomarkers, but also reveal complementary information [11, 21, 22]. When combining two or more biomarkers simultaneously for prediction of AD dementia from MCI, i.e., using MRI and CSF [23, 24], MRI and cognitive testing [25, 26], fluorodeoxyglucose positron emission tomography (FDG-PET) and CSF [27], FDG-PET and cognitive testing [28], and MRI, CSF, and FDG-PET [29, 30], the utility of multiple biomarker combinations for prediction of AD dementia from MCI was confirmed. In particular, a recent study with an advanced multimodal classification method revealed that 91.5% of MCI short-term progressors and 73.4% MCI nonprogressors were correctly classified using baseline MRI, FDG-PET, and CSF data [30]. Combining cognitive measures with CSF or volumetric MRI may substantially improve risk prediction. However, increasing the number of biomarker combinations did not lead to an incremental increase in predictive power. A combination of impaired learning ability with medial temporal atrophy was associated with the greatest risk of developing AD dementia from MCI [31].

A prediction of AD dementia in a foreseeable time period, i.e., within 1 or 2 years, appears much more relevant in a clinical perspective than a prediction of dementia in the more distant future, e.g., in 10–20 years. Individuals classified to be at “short-term risk” can receive more active treatment and counselling. Furthermore, the proportion of MCI patients progressing to AD dementia is not constant over time, but is highest during the first years of follow-up and decreases at longer follow-up intervals [32]. Finally, information about long-term risk of AD dementia may be of limited practical value for patients at the end of their lifespan. In subjects with MCI, the effects of cerebral amyloidosis and hippocampal atrophy on the progression to AD dementia differ, e.g., the risk profile is linear with hippocampal atrophy but reaches a ceiling with higher values for cerebral amyloidosis [1]. In subsequent investigations, biomarkers of neuronal injury appeared to best predict AD dementia from MCI subjects at shorter time intervals (1–2 years) in particular [14, 18].

In the present study, we investigated which combination of cognitive markers and biomarkers can best predict progression to AD dementia in order to generate a clinical model to predict the short-term progression to AD dementia applicable to a help-seeking sample of MCI patients. Knowledge on the added value of combining different biomarker modalities for the most efficient prediction of progression to AD dementia in MCI subjects is still limited. In our analysis, we describe the incremental predictive power of an increasing number of combined biomarkers to determine progression to AD dementia from MCI subjects and test the best two- to four-predictor combinations for superiority over each other.

## Methods

### Dementia Competence Network study

The diagnostic and prognostic study of the Dementia Competence Network (DCN) is a prospective multisite longitudinal observational study on memory clinic patients with MCI or early dementia [33]. Thirteen expert memory clinics of German academic hospitals were involved in data collection. Personnel were trained and experienced in clinical research. Further details are available at the DCN website [34]. The DCN study was approved by the Ethics Review Board of the Erlangen medical faculty (coordinating center) and by the Ethics Committees at each individual center, and was conducted in accordance with the Declaration of Helsinki. All patients gave written informed consent to participate. We adhered to the Standards for Reporting Diagnostic accuracy (STARD) statement [35] to optimize generalizability of the prediction model.

### Subjects

Subjects were recruited between May 2003 and November 2007. They were referred to the participating specialist memory clinics for the workup of memory complaints and/or other cognitive deficits. In most centers, a consecutive series of patients was included. Detailed inclusion and exclusion criteria were published earlier [33]. For the present analysis, all patients had MCI at baseline.

A diagnosis of MCI was made on the basis of clinical and neuropsychological data, without reference to CSF or MRI volumetry results (see below). The DCN study deliberately used a broad definition of MCI [5]—complaints of a cognitive deficit and objectified decline of cognitive abilities (more than 1 SD below age- and education-adjusted norms) in at least one of the following domains as evidenced by standardized neuropsychological tests (Consortium to Establish a Registry of Dementia (CERAD) neuropsychological test battery): verbal learning and memory, nonverbal learning and memory, word fluency, naming, visuoconstruction, cognitive speed, or executive function [36]; no or only minor changes in complex activities of daily living (ADL), as demonstrated by a Bayer activities of daily living (B-ADL) score < 4 [37]; no major depressive episode at baseline as demonstrated by a Montgomery-Asberg Depression Rating Scale score < 13 [38].

With this diagnostic procedure, 1071 MCI patients were included at baseline from which we selected a subsample of 115 (12%) patients according to the following inclusion/exclusion criteria: availability of 1) complete baseline neuropsychological data; (2) adequately processed and quality checked MR imaging; and (3) CSF data at baseline; (4) all had been followed for at least 12 months (mean follow-up 25.46 months) and were clinically evaluated every 12 months up to 36 months or until progression to incident dementia; and 5) outcome—MCI stable or progression to AD only. The major focus of our research was on early prediction of AD from normal. Since our diagnoses are based on clinical classification at follow-up and are not pathological diagnoses, an inclusion of subjects with progression to other dementia diagnoses may lead to misclassification. This potential misclassification would decrease the predictive power of the biomarkers to be analyzed. Stable MCI patients were defined as those with no dementia (Clinical Dementia Rating (CDR) < 1), a Mini-Mental State Examination (MMSE) score of 24 or higher at last follow-up visit, and a B-ADL score < 4 at each follow-up.

Dementia was defined as a clinical diagnosis with cognitive impairment in two or more cognitive domains severe enough to interfere with normal functioning in the community. From a detailed medical history, clinical, neurological, and psychiatric investigation, other reasons for an impaired cognitive performance and performance in ADL were excluded. At follow-up, progression to dementia was defined as newly occurring impairments in instrumental or basic activities of daily living (B-ADL score > 4) in subjects previously defined as having MCI. Patients who developed a non-AD dementia at follow-up were excluded from the analyses.

### Neuropsychological procedures

All subjects were investigated with standardized diagnostic procedures. Neuropsychological analysis was performed as described previously [33]. The following raw memory scores were part of the test battery series at baseline: the CERAD neuropsychological test battery includes a word list learning subtest. A 10-word list is read and immediately recalled three times. After a distraction period of 15 min, while other neuropsychological tests were done, subjects have to recall the items presented and then to recognize them among distractors. We here focused on the delayed free-recall (CERAD-DR) measure. As a composite measure of overall dementia severity, the CDR sum-of-boxes (CDR-sb) was applied [39]. For comparison to other cohorts, the MMSE was applied [40].

### Collecting, storage, and shipment of the samples

Before starting collection of human body fluid samples, standard operating procedures (SOPs) were implemented [41]. Briefly, CSF was collected by lumbar puncture from the L3/L4 or L4/L5 intervertebral region. No serious adverse events were reported. CSF was sampled in polypropylene test tubes with intermediate storage at site (–80 °C), and was then shipped on dry ice to the central biobank (Erlangen University) without undergoing any thawing/refreezing cycles.

### Analyses of CSF biomarkers

The following CSF biomarkers were measured by enzyme-linked immunosorbent assay (ELISA): amyloid-beta_1-40_ (Aβ40; The Genetics Co., Zürich, Switzerland), Aβ42, total tau (t-Tau), and phosphorylated tau^181^ (p-Tau; Innogenetics, Ghent, Belgium). The analyses were performed by experienced laboratory technicians in a certified laboratory and under a routine quality control regime (intra-assay coefficients of variation: 2.3–5.9%; interassay coefficients of variation: 9.8–13.7%). The technicians were blinded to the clinical diagnoses and other clinical information. In addition to Aβ42, several other Aβ isoforms are excreted into the CSF, the most abundant being Aβ40 [42]. CSF Aβ40 is relatively unchanged in AD, but the CSF Aβ42/Aβ40 ratio (Aβ ratio) has been suggested to have stronger diagnostic accuracy for AD compared to CSF Aβ42 alone [43]. The ratio may normalize individuals according to their Aβ production level, so that pathologically low CSF Aβ42 can be identified in “high Aβ producers” and vice versa [44]. Since it has been shown that the Aβ ratio is useful in a clinical setting [45], we also evaluated the predictive value of this ratio.

### Hippocampal volume

MRI scans were obtained on 1.5-Tesla scanners. Special measures were taken for standardization of MRI acquisition across centers. Acquisition parameters were provided to all centers as a guideline. The phantom test of the American College of Radiology MRI Accreditation Program was conducted repeatedly at 11 sites of the DCN [46]. Furthermore, a single volunteer was investigated at each of these centers. Hippocampal volume (HCV) was calculated as the mean value of the left and right hemisphere, and was determined from high-resolution structural magnetic resonance images using the Oxford Centre for Functional MRI of the Brain (FMRIB) Integrated Registration and Segmentation Tool [47] from the FMRIB Software Library (FSL) package of tools [48], since a biological relevance for using the hippocampal volumes of the left and right hemispheres separately cannot be assumed.

Estimated total intracranial volume was calculated through registration of each MRI scan to a standard brain image template [49] using FSL FLIRT [50]. Extensive quality control analysis was performed on segmentations of all volumetric measures. Outliers were visually evaluated by overlaying the automated segmentations on the original MRI scan. Subjects were excluded from the analysis if structures were poorly segmented.

### Outcome measure and predictors

Clinical protocols were uniform across centers and strict SOPs were implemented before recruitment of patients. The “reference standard” was a clinical diagnosis of probable AD dementia in a patient with previous MCI [5]. AD dementia was diagnosed according to the NINCDS-ADRDA criteria [51]. All clinicians who collected follow-up data or who made the diagnoses were blinded to the results of CSF analysis and hippocampal volumetry.

The following measures were evaluated as predictors for progression to AD dementia: MMSE, CDR-sb, CERAD-DR, HCV, Aβ42, Aβ ratio, t-Tau, and p-Tau.

Following the recommendations in the STARD criteria for studies of diagnostic accuracy [35], the cut-off values for defining abnormal biomarker values (HCV and CSF parameters) were developed in subjects that were not part of this study. In the context of this study, percentage abnormal values in relation to these cut-offs were used for descriptive purposes only. Aβ42 (600 pg/ml), p-Tau (60 pg/ml), and total tau (300 pg/ml) cut-offs were provided by the assay producer (Innogenetics, Ghent, Belgium). For the CERAD-DR score, a published cut-off value was used which separated normal controls from MCI (<7) [52].

### Statistical analysis

All statistical analyses were performed using the statistical software R (version 2.3.1) [53]. A correction for multiple testing was not performed due to the low number of tests. Group comparisons at baseline were performed by the Wilcoxon rank sum test for continuous and nominal variables. CSF biomarkers, hippocampal volumes, and neuropsychological data as well as demographic data obtained in MCI patients at baseline were used to predict progression to AD dementia (MCI-AD) versus nonprogression (MCI-stable). We did not control for possible center effects, MCI subtype, or length of follow-up. The predictive power of each single parameter was evaluated by receiver operating characteristic (ROC) area under the curve (AUC) analysis. Sensitivity, specificity, and Youden’s index (sensitivity + specificity – 1) [54] of the various predictors/prediction models were also calculated to provide a complete description of the prediction parameters.

For comparison of the AUC values of single predictors we used a bootstrapping algorithm as implemented in the pROC R-package (Version 1.8) [55]. The predictive accuracy of all possible combinations of two, three, and four parameters was analyzed by a classification system that consisted of a 0.632 bootstrapping wrapper (100 replications) around a support vector machine (SVM) with linear kernel. Whether the addition of a variable significantly increased predictive quality of models with one, two, three, or four predictors was tested by comparing the AUC distributions of 1000 bootstrapping replications for the different classifiers according to Hanley and McNeil [56]. In each bootstrap trial, the different models were ranked according to the AUC. For statistical testing, we calculated a *Z*-score which approximately follows a standard normal distribution.

$$ \mathrm{Z}\frac{mean\left({\uptheta}_1-{\uptheta}_2\right)}{sd\left({\uptheta}_1-{\uptheta}_2\right)} $$

The *p* value was then calculated according to the standard normal distribution. To demonstrate the normality of the *Z* values, a QQ Norm plot was calculated.

As a second way to compare the ROC curves, we evaluated the ROC curves at a given sensitivity of 85%. This is consistent with international recommendations for a biomarker, since values above 80% are considered indicative of satisfactory predictive performance [57]. Using 100 bootstrapping replications, we obtained a distribution of specificities around a given sensitivity of 85%. To compare the ROC curves at the given sensitivity, we used the same procedure as described above to compare the AUCs.

## Results

**Fig. 1:**
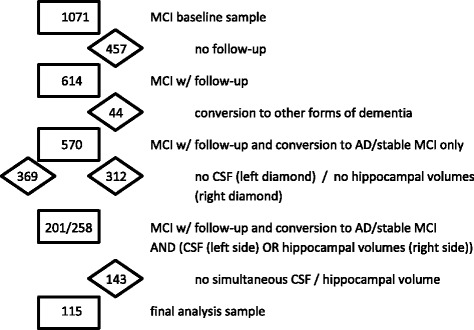
Patient loss due to missing data. The absolute sample size is given in the *rectangles*; the loss of sample size due to missing data of the respective measures is given in the *diamonds. AD* Alzheimer’s disease, *CSF* cerebrospinal fluid, *MCI* mild cognitive impairment

We analyzed 115 patients with complete datasets at baseline and clinical follow-up. The datasets analyzed in the current study are available from the corresponding author on reasonable request. They were a subset from 1071 MCI subjects in whom baseline demographics and neuropsychological test results were available. Due to various missing data, 956 subjects could not be analyzed (see Fig. 1 for exact patient loss). Table 1 compares the demographic characteristics, cognitive and psychometric test scores, hippocampal volume measures, and cerebrospinal fluid biomarkers at baseline between the maximal MCI sample, in those for which the respective measures were available, and the final analysis set (115 patients, 12% of the MCI cohort) which was used for predictor analysis. There was no significant difference between the groups on any parameter (with the exception of CDR-sb which was not clinically relevant). All *p* values were based on pairwise comparisons, uncorrected for multiple testing. Table 2 summarizes the demographic characteristics, cognitive test scores, hippocampal volume measures and cerebrospinal fluid biomarkers at baseline and follow-up for the groups used for the prediction analysis (final analysis set, MCI-AD, MCI-stable) and the statistical differences between the MCI-AD and MCI-stable groups. Of the 115 MCI patients, 28 patients (24.3%) progressed to AD dementia (MCI-AD) after a mean follow-up of 26.2 months corresponding to an annual conversion rate of 11.2%; 87 patients did not progress to AD (MCI-stable), and their mean follow-up was 25.2 months which was not significantly different from the MCI-AD follow-up (Wilcoxon test, *p* > 0.1). In addition, 17 MCI patients progressed to non-AD dementia. Because potential misclassification between clinical diagnosis and actual pathology may decrease the predictive power of the biomarkers to be analyzed, we decided to exclude MCI subjects with clinical progression to non-AD dementias from our analysis. At the first follow-up (year 1), 21 out of 28 MCI subjects had progressed to AD dementia, 5 out of the remaining 7 MCI subjects had progressed to AD dementia at the second follow-up (year 2), and the final 2 MCI subjects had progressed to AD dementia at the third follow-up (year 3).

**Table 1:** Sociodemographic, clinical, and biomarker variables in the final analysis set (*n* = 115) and in the respective comparison group (max. *n* = 956; actual group size for each variable is the maximal *N* available for the comparison group)

Variable	Final analysis sample (*n* =115) MCI-stable + MCI-AD	Comparison group (*n* = 956)		*P* value
		Group size (*n*)	All other MCI with data available	
Age	65.7 ± 9.0	956	67.1 ± 8.6	ns
Sex (male = 1)	1.4 ± 0.5	956	1.5 ± 0.5	ns
education	9.5 ± 1.9	956	9.5 ± 1.9	ns
MMSE	27.0 ± 2.1	956	27.2 ± 2.2	ns
MADRS	7.4 ± 5.6	908	7.8 ± 6.2	ns
B-ADL	2.5 ± 1.5	903	2.3 ± 1.4	ns
CDR-sb	1.8 ± 1.1	956	1.5 ± 1.0	0.01
CERAD-DR-WL	4.8 ± 2.3	956	5.0 ± 2.3	ns
Hippocampal volume	4450 ± 672	368	4511 ± 653	ns
Aβ42	749 ± 300	292	751 ± 353	ns
Aβ40	9654 ± 2732	268	9684 ± 3030	ns
Aβ ratio	0.08 ± 0.03	266	0.08 ± 0.04	ns
Total tau	411 ± 251	284	446 ± 304	ns
Phosphorylated tau	61 ± 30	289	67 ± 36	ns

Data are given as mean ± standard deviation

There is no significant difference between the groups with the exception of CDR-sb which is not clinically relevant

*P* values were uncorrected for multiple comparisons

*Aβ40* amyloid-beta_1–40_, *Aβ42* amyloid-beta_1–42_, *AD* Alzheimer’s disease, *B-ADL* Bayer activities of daily living, *CDR-sb* Clinical Dementia Rating–sum-of-boxes, *CERAD-DR-WL* Consortium to Establish a Registry of Dementia–delayed recall word list, *MADRS* Montgomery-Asberg Depression Rating Scale, *MCI* cognitive impairment, *MMSE* Mini-Mental State Examination, *ns* not significant

**Table 2:** Demographic characteristics, cognitive test scores, APO E allele distribution, brain volumetric measures, and cerebrospinal fluid biomarkers at baseline and follow-up for the final analysis set (115 MCI subjects) and the two groups MCI-stable and MCI-AD

	All (*n* = 115)	MCI-stable (*n* = 87)	MCI-AD (*n* = 28)	Standard mean difference*	*P* value**
Age (years)	65.7 ± 9.03 (36–89)	66.5 ± 8.95 (51–80)	65.4 ± 9.37 (36–89)	0.12	ns
Education (years schooling)	9.50 ± 1.91 (7–13)	9.75 ± 1.95 (7–13)	8.75 ± 1.58 (7–13)	–0.52	<0.05
Gender (female = 1, male = 2)	Male = 67; female = 48	Male = 52; female = 35	Male = 15; female = 13	0.13	ns
Bayer-ADL scale (score: 1–10)	2.47 ± 1.48 (1–4)	2.41 ± 1.52 (1–4)	2.67 ± 1.34 (1–4)	0.18	ns
MADRS (score: 0–60)	7.41 ± 5.75 (0–13)	7.67 ± 5.96 (0–13)	6.62 ± 5.08 (0–11)	–0.18	ns
MMSE	27.0 ± 2.12 (20–30)	27.5 ± 1.87 (22–30)	25.8 ± 2.34 (20–29)	–0.81	<0.001
CDR-sb	1.80 ± 1.06 (0.5–4.5)	1.59 ± 1.00 (0.5–4.5)	2.45 ± 0.98 (0.5–4)	0.81	<0.001
CERAD-DR-WL	4.82 ± 2.33 (0–10)	5.25 ± 2.23 (0–10)	3.46 ± 2.15 (0–8)	–0.77	0.001
Amnestic deficit: CERAD-DR-WL below cut-off (<7 correct responses), *n* (% present)	88 (77%)	62 (71%)	26 (93%)		
ApoE4 alleles (homo- or heterozygotes), *n*/sample size (% present)	41/103 (40%)	32/78 (41%)	9/25 (36%)		ns
Hippocampal volume (mm^3^)	4450 ± 672 (2509–5996)	4585 ± 649 (3036–5996)	4031 ± 570 (2509–5235)	–0.82	<0.0001
Total tau in CSF (pg/ml)	411 ± 252 (112–1169)	351 ± 205 (112–1158)	596 ± 294 (156–1169)	0.97	<0.0001
Total tau in CSF below cut-off (>300 pg/ml), *n* (% abnormal)	65 (57%)	41 (47%)	24 (86%)		
Phosphorylated tau in CSF (pg/ml)	61.3 ± 30.5 (19.7–157)	55.6 ± 26.3 (19.7–130)	78.8 ± 36.1 (27.3–157)	0.76	<0.01
Phosphorylated tau in CSF below cut-off (>60 pg/ml), *n* (% abnormal)	44 (38%)	27 (31%)	17 (61%)		
Aβ42 in CSF (pg/ml)	749 ± 300 (245–1792)	794 ± 309 (276–1792)	611 ± 223 (245–1134)	–0.61	<0.001
Aβ42 in CSF below cut-off (<600 pg/ml), *n* (% abnormal)	39 (34%)	22 (25%)	17 (61%)		
Aβ40 in CSF (pg/ml)	9654 ± 2731 (2604–16320)	9601 ± 2561 (4175–16320)	9817 ± 3251 (2604–15210)	0.08	ns
Aβ40 in CSF below cut-off, *n* (% abnormal)	n/a	n/a	n/a		
Follow-up time (months)	25.5 ± 9.8 (12–36)	26.1 ± 8.0 (12–36)	25.2 ± 8.9 (12–36)		ns

Values are given as means ± SD (range) unless otherwise stated

*Standardized (mean values in MCI-AD patients – mean values in MCI-stable patients)/standard deviation in the group of all patients

**P values refer to differences between MCI-stable and MCI-AD

*Aβ40* amyloid-beta_1–40_, *Aβ42* amyloid-beta_1–42_, *AD* Alzheimer’s disease, *B-ADL* Bayer activities of daily living, *CDR-sb* Clinical Dementia Rating–sum-of-boxes, *CERAD-DR-WL* Consortium to Establish a Registry of Dementia–delayed recall word list, *CSF* cerebrospinal fluid, *MADRS* Montgomery-Asberg Depression Rating Scale, *MCI* cognitive impairment, *MMSE* Mini-Mental State Examination, *n/a* not available, *ns* not significant

### Baseline characteristics

Both MCI subgroups were well matched in terms of age and gender, and showed a similar distribution of APO E alleles (Table 2). MCI patients progressing to AD (MCI-AD) had fewer years of schooling, lower MMSE and CERAD-DR scores, higher CDR-sb scores, and presented with significantly higher t-Tau and p-Tau values, significantly lower mean Aβ42 levels in CSF, and significantly smaller HCV. The standardized mean difference (SMD) at baseline was largest for the t-Tau values, followed by HCV. Among the biomarkers analyzed, Aβ42 levels showed the smallest SMD at baseline.

Regarding the frequency of abnormal values, 86% of MCI-AD patients had abnormal values for t-Tau, 61% for p-Tau, and 61% for Aβ42. The MCI-stable subgroup had considerably lower frequencies of abnormal biomarker values: 47% for t-Tau, 27% for p-Tau, and 25% for Aβ42.

### Prediction of incipient AD

ROC curve analysis resulted in AUC of 0.71 to 0.77 for the biomarkers t-Tau and HCV and the cognitive markers CDR-sb, CERAD-DR, and MMSE. AUC > 0.7 corresponds to a fair accuracy for predicting progression to AD dementia. p-Tau, Aβ42, and Aβ ratio had an AUC < 0.7 and, thus, poorly predicted progression (Table 3). Interestingly, the sensitivities and specificities of the individual predictors were inversely related to AUC values, possibly reflecting the effect of the short-term follow-up period. Pairwise comparisons of the single biomarker predictors showed all AUC curves were significantly different from Aβ40 (*p* < 0.05), but not from each other with the one exception that t-Tau was significantly different from p-Tau (*p* < 0.03) (see Table 4).

**Table 3:** The 10 single predictors and the five best two- to four-predictor classification results predicting progression from MCI to AD dementia

Predictors				AUC	Youden index	Sensitivity	Specificity	PPV	NPV
*Single predictors*									
t-Tau				0.77	1.48	0.62	0.86	0.42	0.93
HCV				0.74	1.45	0.63	0.82	0.42	0.92
CDR-sb				0.73	1.38	0.45	0.93	0.35	0.95
CERAD-DR				0.72	1.39	0.64	0.75	0.40	0.89
MMSE				0.71	1.36	0.68	0.68	0.40	0.87
p-Tau				0.69	1.41	0.81	0.61	0.50	0.86
Aβ42				0.68	1.38	0.74	0.64	0.44	0.87
Aβ42/Aβ40				0.66	1.34	0.59	0.75	0.37	0.88
Aβ40				0.55	1.15	0.76	0.39	0.34	0.80
APO E				0.50	1.05	0.41	0.64	0.26	0.47
*Two-predictor combinations*									
t-Tau	HCV			0.81	1.45	0.68	0.77	0.49	0.88
t-Tau	CDR-sb			0.79	1.44	0.83	0.61	0.41	0.92
t-Tau	MMSE			0.79	1.46	0.81	0.65	0.43	0.91
t-Tau	CERAD-DR			0.78	1.43	0.62	0.81	0.52	0.87
p-Tau	HCV			0.77	1.40	0.76	0.64	0.42	0.89
*Three-predictor combinations*									
t-Tau	HCV	CDR-sb		0.83	1.54	0.84	0.70	0.48	0.93
t-Tau	HCV	MMSE		0.81	1.47	0.83	0.64	0.44	0.92
t-Tau	MMSE	CDR-sb		0.81	1.47	0.76	0.70	0.46	0.90
t-Tau	CDR-sb	CERAD-DR		0.80	1.48	0.79	0.70	0.47	0.91
HCV	MMSE	Aβ42/Aβ40		0.80	1.48	0.78	0.70	0.45	0.91
*Four-predictor combinations*									
t-Tau	HCV	CDR-sb	Aβ42/Aβ40	0.82	1.55	0.89	0.67	0.47	0.95
t-Tau	p-Tau	CDR-sb	Aβ42/Aβ40	0.82	1.49	0.78	0.71	0.47	0.91
t-Tau	HCV	p-Tau	Aβ42/Aβ40	0.82	1.49	0.85	0.64	0.42	0.93
t-Tau	HCV	CDR-sb	CERAD-DR	0.81	1.57	0.80	0.76	0.52	0.92
HCV	MMSE	CDR-sb	Aβ42/Aβ40	0.81	1.51	0.86	0.65	0.46	0.93

The predictors are sorted according to the AUC

All values correspond to mean bootstrap-based cross-validated performance

*Aβ40* amyloid-beta_1–40_, *Aβ42* amyloid-beta_1–42_, *AD* Alzheimer’s disease, *AUC* area under the curve, *CDR-sb* Clinical Dementia Rating–sum-of-boxes, *CERAD-DR* Consortium to Establish a Registry of Dementia–delayed recall, *HCV* hippocampal volume, *MCI* cognitive impairment, *MMSE* Mini-Mental State Examination, *NPV* negative predictive value, *PPV* positive predictive value, *p-Tau* phosphorylated tau, *t-Tau* total tau

**Table 4:** *P* values showing pairwise statistical comparisons of single predictor receiver operating characteristic curves based on area under the curve

	HCV	Aβ42	Aβ40	p-Tau	t-Tau	MMSE	CDR-sb	CERAD-DR	Aβ42/Aβ40
HCV		ns	**0.03**	ns	ns	ns	ns	ns	ns
Aβ42			ns	ns	ns	ns	ns	ns	ns
Aβ40				**0.02**	**0.00**	**0.04**	**0.02**	**0.05**	0.06
p-Tau					**0.03**	ns	ns	ns	ns
t-Tau						ns	ns	ns	**0.02**
MMSE							ns	ns	ns
CDR-sb								ns	ns
CERAD-DR									ns

Significant values are shown in bold typeface

Data were not corrected for multiple testing

For comparing areas under the curve we used a bootstrapping algorithm as implemented in pROC packages (Version 1.8)

*Aβ40* amyloid-beta_1–40_, *Aβ42* amyloid-beta_1–42_, *CDR-sb* Clinical Dementia Rating–sum-of-boxes, *CERAD-DR* Consortium to Establish a Registry of Dementia–delayed recall, *HCV* hippocampal volume, *MMSE* Mini-Mental State Examination, *ns* not significant, *p-Tau* phosphorylated tau, *t-Tau* total tau

### Statistical comparison of the predictive power of biomarker combinations over individual biomarkers

**Fig. 2:**
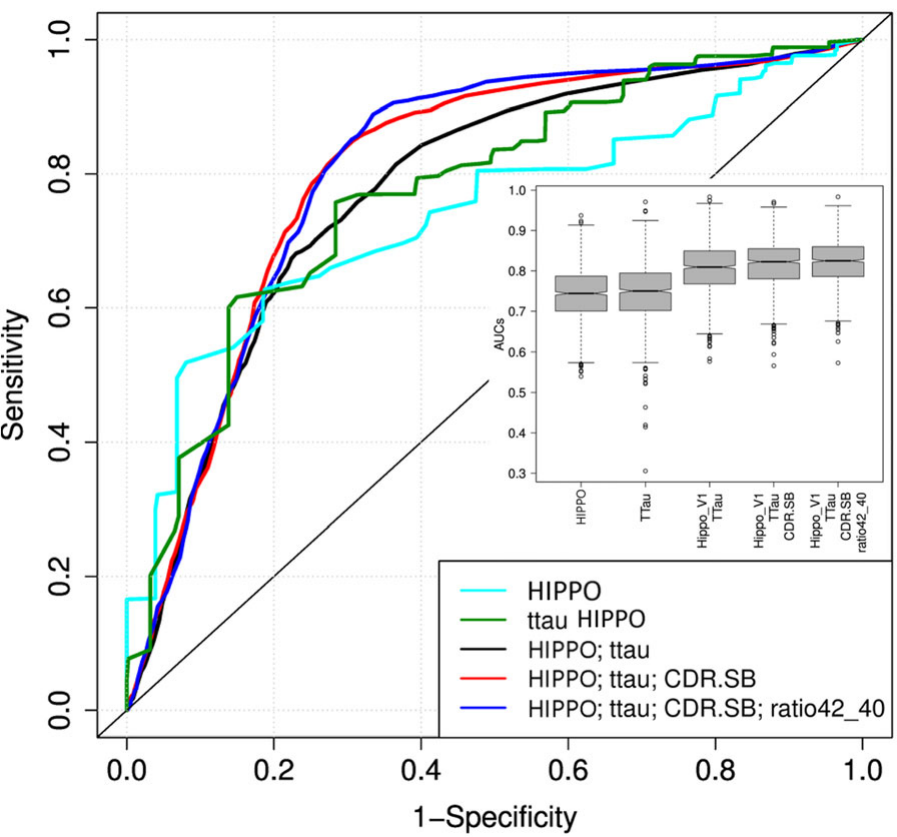
ROC curves of the best single and two- to four-parameter classifiers, based on 100 replicates of a 0.632 bootstrapping algorithm based on support vector machine classifier. A boxplot of areas under the curves (*AUCs*) for the different classification engines is shown in the insert. The AUC values of the different classification engines were not significantly different from each other. *CDR.SB* Clinical Dementia Rating–sum-of-boxes, *HIPPO* hippocampal volume, *ttau* total tau, *ratio42_40* amyloid-beta_1–42_/amyloid-beta_1–42_ ratio

To test if a combination of the best two- to four-biomarker combinations outperforms the two best individual biomarkers in separating progressive MCI (i.e., those MCI subjects who progressed to AD dementia during the follow-up period (MCI-AD)) from stable MCI, the analysis of two- to four-parameter combinations of the eight predictor/biomarker indices (MMSE, CDR-sb, CERAD-DR, HCV, Aβ42, Aβ42/Aβ40, t-Tau, and p-Tau) were numerically superior over the performance of a single biomarker index. Classification results for prediction of progression from MCI to AD dementia (MCI-AD) of the best one- to four-parameter predictor combinations are given in Table 3. t-Tau was the single marker with the highest mean bootstrap-based cross-validated AUC of 0.77, followed by HCV (AUC = 0.74). Compatible with this, the best two-predictor combination was t-Tau and HCV (AUC = 0.81). The addition of a third and fourth predictor only minimally increased the mean bootstrapping-based cross-validated AUC (the best three-predictor combination was t-Tau/HCV/CDR-sb, AUC = 0.83; the best four-parameter combination was t-Tau/HCV/CDR-sb/Aβ ratio, AUC = 0.82). The pairwise comparisons of AUC values for HCV or t-Tau with the best two-, three- or four-predictor combinations were not significantly different. The AUC-curves for the best one to four predictor(s) combinations are illustrated in Fig. 2. When MCI subjects with progression to non-AD dementia were included in our analysis (e.g., added to the MCI-stable group), the results remained largely unchanged for the two- and three-parameter combinations, but varied in the four-parameter combinations (data not shown).

**Fig. 3:**
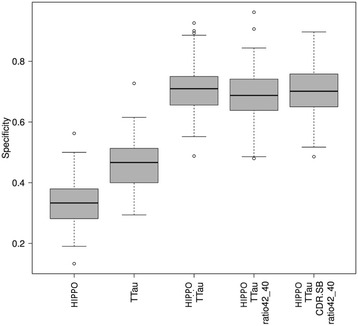
Boxplot of specificities at a given sensitivity of 85% for the different predictor combinations. AUC were calculated by 100 replications of a .632 bootstrapping algorithm. p-values for comparing specificities at the given sensitivity demonstrated the single value predictors HCV and total tau were significantly inferior compared to the two-four predictor combinations. None of the predictor combinations were significantly different from each other. *CDR.SB* Clinical Dementia Rating–sum-of-boxes, *HIPPO* hippocampal volume, *ttau* total tau, *ratio42_40* amyloid-beta_1–42_/amyloid-beta_1–42_ ratio

Finally, we compared the normalized distribution of bootstrapped specificities at a fixed sensitivity of 85% for the individual biomarkers HCV and t-Tau and the best two- to four-parameter combinations. Here, the statistical comparison of specificities at the fixed sensitivity of 85% revealed that all two- to four-parameter combinations were significantly superior to HCV, but only the three/four-parameter combinations were superior to t-Tau alone (*p* < 0.03, uncorrected for multiple comparisons). When comparing the best two- to four-parameter combinations among each other, there were no significant differences (HCV/t-Tau, HCV/t-Tau/CDR-sb, HCV/t-Tau/CDR-sb/Aβ ratio) (*p* > 0.10, uncorrected for multiple comparisons). QQ plots of standardized specificities at the given sensitivity of 86% confirmed their standard normal distribution (see Table 5 and Fig. 3).

**Table 5:** *P* values showing statistical comparisons of the specificities of the receiver operating characteristic curves for the best one-, two-, three-, and four-predictor combinations at a given sensitivity of 85%

	t-Tau	t-Tau, HCV	t-Tau, HCV, CDR-sb	t-Tau, HCV, CDR-sb, Aβ42/Aβ40
HCV	ns	0.03	0.001	0.000
t-Tau		ns	0.01	0.003
t-Tau, HCV			ns	ns
t-Tau, HCV, CDR-sb				ns

Data were not corrected for multiple testing

For comparing the values we used a bootstrapping algorithm as implemented in pROC packages (Version 1.8)

All predictor combinations were statistically superior to single predictors, but none of the best three- and four-parameter combination was statistically superior to the two-predictor combination

*Aβ40* amyloid-beta_1–40_, *Aβ42* amyloid-beta_1–42_, *CDR-sb* Clinical Dementia Rating–sum-of-boxes, *HCV* hippocampal volume, *ns* not significant, *t-Tau* total tau

## Discussion

The power of hippocampal volume, CSF Alzheimer biomarkers, and neuropsychological measures for predicting progression from MCI to AD dementia was analyzed in a relatively large multicentre memory clinic cohort from the German Dementia Competence Network (DCN). A combination of two biomarkers of neurodegeneration (e.g., HCV and t-Tau) did not predict AD dementia in MCI significantly better than any parameter alone, and none of the possible three- to four-parameter combinations improved the predictive power. Our study is unique in applying advanced statistical methods for testing different biomarker combinations for superiority over each other.

A systematic incremental combination of the nine independent predictor variables in models with maximally four predictors allowed for the direct comparison of single predictor models with more complex models, based upon a bootstrapping algorithm for the AUC. There was a numerical gain by up to 6% in AUC from the best single-predictor model to the best four-predictor model, but none of the differences between one-predictor models and the best two-predictor model were statistically significant. Only when specificities were compared for a given sensitivity of 85% was there an inferiority of hippocampal volume to the best two- to four-parameter combinations. Although it is possible that a test of these models in a larger sample or a combination of even more predictors may lead to a generally superior multipredictor model, the current results support the assumption that an easy to use and economic one-predictor model of neurodegeneration markers may be as good as more complex models for the prediction of progression from MCI to AD dementia within a relatively short time interval of 2 years. Indeed, due to typically high collinearity of predictor variables, more complex models are not likely to increase prediction accuracy [58].

Our cohort of MCI patients is generated from a large multicenter study of German university memory clinics in patients with various degrees of cognitive impairment ranging from subjective cognitive decline to various forms of dementia which differs, for example, from the Alzheimer’s Disease Neuroimaging Initiative (ADNI) cohort in terms of setting and recruitment [33]. A selection bias of the final analysis set of moderate sample size from the much larger total MCI cohort could not be demonstrated in our data. MCI participants in ADNI were carefully selected to include individuals with documented memory impairment and to exclude those whose impairment could arise from other potential causes. Thus, the ADNI MCI population is not representative of a general clinical population, but more of a research population. In our sample, the inclusion and exclusion criteria were more liberal [33] as the patients were recruited from a help-seeking clinical sample. Also, the neuropsychological criteria to diagnose MCI used a more liberal threshold to define cognitive impairment, following the criteria proposed by Winblad et al. [5]. The mean age of our sample (66 years) was considerably younger than in ADNI (74 years) [59], and in the DESCRIPA study (71 years) [60]. This may impact on the proportion of subjects progressing to AD dementia at the given follow-up rate. Clinically, 24.3% of the MCI patients of the DCN cohort progressed to AD dementia after a mean follow-up period of 25.5 months, corresponding to an annualized conversion rate of 12%. This rate is lower than an approximate 30% progression rate in other multicenter cohorts with comparable follow-up (32% DESCRIPA [60, 61], 30–44% ADNI [24, 31, 59], and 30% AIBL [62]). After a mean follow-up period of 3.3 years, 44.6% of MCI subjects from ADNI had progressed to AD dementia [59], in line with the notion that the likelihood of progression to AD dementia increases with follow-up time. In summary, the lower progression rate may be attributed to the lower mean age, a broader definition of MCI, and differences in recruitment strategy of patients in our sample compared to other cohorts with similar follow-up time [62–64].

As in other studies, the groups of MCI-stable and MCI-AD were not different with respect to age, education, gender, APO E genotype, and Aβ40, and all baseline Alzheimer biomarker levels were pathologically changed in MCI-AD compared to MCI-stable. With respect to their predictive power, a shorter follow-up time generally favors biomarkers of neurodegeneration, e.g., tau and HCV, since neurodegeneration occurs shortly before (or even triggers) progression from MCI to dementia stage. In univariate analyses, the biomarkers t-Tau and HCV and the cognitive markers CDR-sb, CERAD-DR, and MMSE fairly well predicted progression to AD dementia, while p-TAU, Aβ42, and Aβ40 only poorly predicted progression. APO E genotype did not have any predictive value. None of the single predictors reached the criterion of an AUC > 0.8 for a diagnostic biomarker, suggested by a Consensus report [57]. Almost all of the two- to four-predictor combinations increased the predictive power over the threshold AUC > 0.8, but none of the three- and four-predictor combinations were significantly superior to the two-predictor combination of the injury markers HCV and t-Tau. Similar to our data, data from ADNI showed that a combination of a measure of volumetric change and t-Tau in CSF was associated with higher risk of progression to AD dementia from MCI compared with each marker alone [24]. In the European multicentre DESCRIPA study, these injury markers predicted time to dementia in subjects with MCI and proven amyloid pathology [65].

Diagnostic biomarkers for the cross-sectional diagnosis of AD dementia compared to controls should have a sensitivity and specificity above 80% [57]. Our best two- and three-predictor combinations were both above this criterion for sensitivity but not for specificity and had a Youden’s index > 1.45. This relative lack of specificity may mainly be due to two reasons: 1) despite extensive inclusion and exclusion criteria, there will be some heterogeneity of underlying disease in MCI patients; 2) at a mean follow-up interval of approximately 2 years (maximum 3 years), some patients classified as MCI-stable will not yet have progressed to AD dementia. This hypothesis is consistent with findings of a meta-analysis showing a trend towards increasingly higher effect sizes of CSF biomarkers including t-Tau, p-Tau and Aβ42 during longer clinical follow-up [66]. In conclusion, the distinction of clinical entities (healthy versus diseased) differs between a short-term prediction of progression to AD dementia as compared to a cross-sectional diagnosis of AD dementia.

Three aspects of the data on individual predictors deserve comment. Firstly our data are based on a variable length of follow-up (1–3 years) which optimizes the available study information. A homogeneous follow-up of, for example, 1 year resulted in lower predictive values due to a reduced number of subjects progressing to AD dementia (21 instead of 28 patients). Hippocampal volume and total tau remained the best single predictors and two-parameter combinations. Furthermore, the order of the best three-predictor combination also remained unchanged (data not shown). Thus, any restriction to a homogenous short follow-up time will underestimate the predictive power of all parameters. Scientifically, a follow-up time until death of study participants would be desirable, but this would be accompanied by considerable drop-out due to various age- and disease-associated reasons. From a clinical standpoint, a follow-up time of 2–3 years may be an acceptable compromise to inform patients with MCI about their foreseeable future, rather than a follow-up time of, for example, 10 years or more, because their life expectancy may be limited. Also, the inclusion of 17 non-AD converters into the MCI-stable group did not impact our analysis. This suggests that our model could apply to unselected MCI subjects, although the large drop-out rate due to loss to follow-up and missing biomarker information limits the generalizability. Secondly, with respect to CSF analyses, it has been argued that a lumbar puncture (LP) for CSF protein analysis may be too invasive for routine use in patients with dementia or MCI. Although there are some contraindications to LP, several studies have shown negligible frequency of complications, especially in the elderly. This supports a routine analysis of CSF biomarkers as part of the clinical diagnostic workup of patients with cognitive impairment possibly due to AD [67]. Thirdly, Aβ42 in CSF was abnormal in just 34% of the total MCI sample. In the MCI-AD subgroup, which consisted largely of patients with amnestic MCI (26/28 patients), roughly two-thirds of the patients had abnormal Aβ42 values. The remaining third of patients would have to be classified as suspected non-AD pathophysiology (SNAP), at a frequency roughly consistent with a previous review [68]. Again, our sample of patients is more representative of a general help-seeking memory clinic population, and a greater heterogeneity of underlying disease in MCI patients is to be expected as compared to a research population. Also, Aβ42 in CSF was the individual parameter least predictive for progression to AD dementia. In a previous publication on MCI patients from the DCN [69], episodic memory measures were highly significantly related to a CSF AD+ signature (Hulstead score, which includes measures of Aβ42 and t-Tau [70]). This suggests that our sample does contain an enriched sample of patients with incipient Alzheimer pathology.

However, at the short follow-up interval of approximately 2 years (maximum 3 years), some patients classified as MCI-stable will not yet have progressed to AD dementia and biomarkers of neurodegeneration will be favored over biomarkers of amyloid pathology in predicting progression to AD dementia. This assumption is supported by the poorer specificity of Aβ42 compared to t-Tau (0.64 versus 0.86), which reflects a lower ratio of negative cases (i.e., such MCI subjects that will remain stable over a much longer observation period), whereas the sensitivity (a ratio of such MCI cases that characterize with pathological biomarkers and have already progressed to dementia) of Aβ42 is superior to t-Tau (0.74 versus 0.62) and slightly superior to hippocampal volume (0.71). Comparable data were obtained by Davatzikos et al. [71] in a subset of patients for whom both CSF and spatial patterns of brain atrophy AD score (SPARE-AD) values were available. However, some further analyses of ADNI data with longer follow-up [59] and DESCRIPA data [61] are in disagreement with the relative superiority of CSF t-Tau over CSF Aβ42 in predictor combinations. It had been suggested that the ratio of Aβ42/Aβ40 may improve the validity of the CSF amyloid AD biomarker for diagnosis [43, 44]. In our dataset, however, the Aβ42/Aβ40 ratio was not consistently superior to Aβ42 alone for predicting AD dementia in MCI patients.

In several analyses using diverse subgroups of MCI patients from the ADNI cohort, several combinations of different biomarkers were tested to predict future cognitive decline or progression to AD dementia; different measures of cortical atrophy including hippocampal volume and, less significantly, the tau/Aβ-42 ratio, predicted cognitive decline in MCI [29]. Landau et al. [11] found that several biomarkers predicted cognitive decline in univariate models, but only reduced glucose metabolism and episodic memory predicted progression to AD. Ewers et al. [59] in their comparison of the effectiveness of single variables and multiple variables in predicting the conversion of MCI to AD found that the best single predictors were comparable in accuracy with the best multiple predictor models, which included right hippocampal volume, CSF p-Tau/Aβ42, TMT-B, and age. Davatzikos et al. [71] reported that the best combination of biomarkers for predicting AD dementia from MCI was a combination of SPARE-AD score, summarizing brain atrophy patterns, with CSF total tau. Gomar et al. [12] found that their most predictive model included two measures of episodic memory and one MR volume measure. An approach using the weighted fusion of data from both high- and low-dimensional modalities [72] found that MCI to AD conversion was optimally predicted by a combination of FDG-PET, MRI shape information, and CSF biomarkers, although CSF biomarkers added only minor improvement. By integrating multimodal data in a probabilistic manner, Young et al. [73] predicted conversion of MCI to AD over 3 years with an accuracy of 72.2%. Heister et al. [31] stratified the ADNI MCI cohort by degree of MR atrophy, CSF biomarker levels, or the degree of learning impairment, and assessed the contribution of each factor to the MCI to AD conversion. Learning impairment plus MR atrophy were associated with the highest risk (HR = 29.0) for conversion. A lack of sensitivity of Aβ42 for prediction was noted. Trzepacz et al. [74] analyzed 29 numeric neuroimaging variables for their performance for predicting conversion from MCI to AD dementia at 2 years. MRI measures had the highest predictive accuracy (67%) which increased (76%) when combined with PIB-PET, producing the highest accuracy among any biomarker combination. For the DESCRIPA cohort, a multicenter European cohort with a 5-year follow-up period, it has been shown that in subjects with MCI and evidence of amyloid pathology, the injury markers CSF t-Tau, p-Tau, and hippocampal atrophy can best predict cognitive decline [60]. Similarly, earlier studies with smaller sample sizes have shown that a combination of hippocampal volume on MRI and CSF-based biomarkers may increase prediction accuracy [23, 75].

### Limitations

The present study has some limitations. Firstly, only patients who were subjected to a CSF analysis at baseline and were able to undergo MRI and who returned for clinical follow-up were analyzed. However, a sampling bias in our final analysis set could not be demonstrated. Secondly, the variability of CSF biomarker levels between laboratories [76–80] makes it difficult to introduce generally applicable cut-off values. To minimize variability we had installed SOPs for pre-analytical sample handling prior to the initiation of the study [41] and analyzed the CSF samples in one centre only. Thirdly, and as in most other predictive studies, patient classification relied on a clinical diagnosis which is not always accurate, especially at early stages of the disease. None of the cases investigated here was neuropathologically verified. This was one of the reasons for excluding patients from our analyses who progressed to other forms of dementia. Fourthly, our study was performed in a memory clinic setting with a tertiary referral structure and therefore should be replicated in other more general settings. This might lead to datasets with a number of incomplete cases. However, the advanced diagnostic procedures (MRI and LP) will be restricted to specialist centers and motivated patients (typical for tertiary referral samples or a memory clinic population) if ever implemented into clinical practice. This may facilitate the generation of complete datasets as used in our study. Furthermore, our statistical methods require complete datasets to allow group comparisons by bootstrapping algorithms. Finally, we applied a relatively short follow-up period with a mean of 2 years, meaning that a certain proportion of patients who were classified as stable are likely to progress to dementia later on. This may explain the low positive predictive value, a value which had not been explicitly referred to before. However, the successful short-term prediction is also a particular strength of the study (see above).

There are several other strengths of the study presented here. It is a large prospective multicentre study investigating the predictive properties of core dementia biomarkers with uniform protocols across centers and with prior installation of SOPs, including the use of appropriate CSF sample tubes and overall standardized pre-analytical CSF handling. The patients were phenotyped and followed-up in expert university memory clinics, which increases the quality of the diagnostic workup. We adhered to the STARD recommendations. All these measures should optimize the generalizability of the results.

## Conclusion

Our results show that a combination of two biomarkers of neurodegeneration (e.g., HCV and t-Tau) is not superior over the single parameters alone in identifying patients with MCI who are most likely to progress to AD dementia within a relatively short time period. However, there is a gradual increase in the statistical measures across increasing biomarker combinations (always involving t-Tau and HCV as the best parameters). From our data it is not possible to deduct recommendations on how to optimize the predictive diagnosis in individual patients with MCI in clinical practice. For enrichment strategies of MCI patients progressing to AD dementia for clinical trials, a combination of two neurodegeneration parameters (HCV and t-Tau) in addition to clinical measures such as CDR-sb may maximize progression rates in order to minimize false negative results of intervention studies.

## Abbreviations

AD: Alzheimer’s disease
ADL: Activites of daily living
ADNI: Alzheimer’s Disease Neuroimaging Initiative
AIBL: Australian Imaging, Biomarkers and Lifestyle
AUC: Area under the curve
Aβ40: Amyloid-beta_1–40_
Aβ42: Amyloid-beta_1–42_
B-ADL: Bayer activities of daily living
CDR: Clinical Dementia Rating
CDR-sb: Clinical Dementia Rating sum-of-boxes
CERAD: Consortium to Establish a Registry of Dementia
CERAD-DR: Consortium to Establish a Registry of Dementia delayed recall
CSF: Cerebrospinal fluid
DCN: Dementia Competence Network
DESCRIPA: Development of screening guidelines and clinical criteria for predementia, Alzheimer's disease
ELISA: Enzyme-linked immunosorbent assay
FDG-PET: Fluorodeoxyglucose positron emission tomography
FLIRT: FMRIB Linear Image Registration Tool
FMRIB: Oxford Centre for Functional MRI of the Brain
FSL: FMRIB Software Library
HCV: Hippocampal volume
LP: Lumbar puncture
MCI: Mild cognitive impairment
MCI-AD: Mild cognitive impairment progressing to Alzheimer’s disease
MMSE: Mini-Mental State Examination
MRI: Magnetic resonance imaging
NINCDS-ADRDA: National Institute of Neurological and Communicative Disorders and Stroke, Alzheimer's Disease and Related Disorders Association
p-Tau: Phosphorylated tau
ROC: Receiver operating characteristic
SNAP: Suspected non-Alzheimer disease pathophysiology
SOP: Standard operating procedure
SPARE-AD score: Spatial patterns of brain atrophy Alzheimer’s disease score
STARD: Standards for Reporting Diagnostic accuracy
SVM: Support vector machine
t-Tau: total tau
